# Metaverse-Based Psychiatric Consultation for Youths With Mental Health Conditions: Qualitative Descriptive Feasibility Study

**DOI:** 10.2196/83688

**Published:** 2026-05-05

**Authors:** Mio Ishii, Junichi Fujita, Nao Toyohara, Keiko Ide, Tomoko Moroga, Mizuho Takayama, Takeshi Asami, Tomoyuki Miyazaki

**Affiliations:** 1 Department of Psychiatry, Yokohama City University School of Medicine, 3-9 Fukuura, Kanazawa-ku, Yokohama, 236-0004, Japan, 81 45-787-2800; 2 Center for Promotion of Research and Industry-Academic Collaboration, Yokohama City University, Yokohama, Japan; 3 Department of Child Psychiatry, Yokohama City University Hospital, Yokohama, Kanagawa, Japan

**Keywords:** mental health, metaverse, youth mental health, virtual reality, telepsychiatry

## Abstract

**Background:**

Youth mental health is a global public health priority, with rising rates of anxiety and depression, particularly after the COVID-19 pandemic. Despite the early onset and substantial burden of mental disorders in this age group, young people are less likely than adults to seek professional help and face barriers such as workforce shortages, stigma, and low mental health literacy. Although efforts such as outreach initiatives and school-based programs have been implemented, innovative and scalable solutions remain limited. Digital technologies, including the metaverse, may offer flexible and stigma-reducing approaches to mental health care; however, evidence regarding their real-world feasibility and acceptability is scarce.

**Objective:**

This study investigated the feasibility and user experience of metaverse-based psychiatric consultations for young people with mental health conditions.

**Methods:**

We conducted a qualitative descriptive feasibility study at a single academic institution in Yokohama, Japan, between July and November 2023. A total of 26 participants aged 16 to 25 years (mean age 19.9, SD 2.4; 15 male, 7 female, 3 nonbinary, 1 no response) who self-identified as having mental health concerns were recruited from local psychiatric clinics, schools, universities, and social media. Reported concerns included anxiety, depressive symptoms, and autism spectrum disorder traits. Participants completed a 30- to 40-minute one-on-one metaverse-based consultation with a psychiatrist using avatars in a virtual reality environment, followed by semistructured interviews exploring feasibility, usability, and user perceptions. Data were analyzed using thematic analysis, and data collection continued until no substantially new themes emerged. Reporting followed the APA Journal Article Reporting Standards for qualitative research.

**Results:**

All participants completed the study without any adverse psychological events. Five participants experienced minor, transient physical discomfort (eg, headaches and virtual reality–related sickness), which resolved without medical intervention. Thematic analysis identified 3 primary domains: perceived psychological safety through avatar-mediated interaction, enhanced spatial presence facilitating rapport, and increased autonomy within the virtual environment. Metaverse consultations were perceived as particularly beneficial for individuals experiencing interpersonal anxiety, sensory sensitivities (including autism spectrum disorder traits), difficulty leaving home due to psychiatric conditions, psychological resistance to traditional psychiatric settings, or discomfort with physical self-presentation.

**Conclusions:**

This qualitative descriptive feasibility study provides preliminary evidence that metaverse-based psychiatric consultations are a feasible and acceptable approach for supporting young people with mental health conditions. Unlike conventional telepsychiatry based on videoconferencing, the use of avatar-mediated interaction and immersive virtual environments may reduce psychological barriers related to self-presentation, stigma, and interpersonal anxiety for specific subgroups of youth. These findings suggest that metaverse-based consultations can be effectively integrated into clinical pathways as a complementary, “low-threshold” access point within stepped or hybrid care models, ultimately bridging the gap between initial help-seeking and formal psychiatric treatment.

## Introduction

Youth mental health is a critical global issue that remains complex and challenging. Reports indicate that 1 in 5 young people experiences anxiety, and 1 in 4 faces depression due to factors such as poverty and shifts in lifestyle patterns [1,2]. This prevalence doubled before and after the COVID-19 pandemic [3]. With more than 25% of mental disorders beginning before the age of 25 years [4] and the significant socioeconomic impact of these conditions [5], the need for effective mental health care for young people is clear. Despite this, young people are less likely to seek professional support for mental health problems than older age groups and often struggle to access timely and high-quality mental health services (MHSs) [6-8]. Several factors contribute to this gap between demand and supply, including physical barriers such as the shortage and geographic maldistribution of child and adolescent psychiatrists, as well as psychological barriers such as stigma and low mental health literacy [9-11]. Although initiatives, including school-based psychoeducation, peer training, and outreach programs, have been implemented to mitigate these barriers, a breakthrough solution remains elusive [6,12-14], underscoring the need for further research.

As an innovative response to these challenges, information and communication technology (ICT) has become increasingly central to youth mental health care. McGorry et al [9] have called for a redesign of youth MHSs and conceptualized youth psychiatry as a distinct discipline, emphasizing early intervention, co-designed youth-friendly services, integration of digital technology with human support, and an extended service boundary up to age 25. Consistent with this framework, ICT-based services are expected to enhance accessibility, reduce stigma, and provide flexible, developmentally sensitive support for late adolescents and young adults, whose daily communication and help-seeking increasingly occur online [9,15]. However, recent systematic reviews caution that the real-world impact of digital youth MHSs may be constrained by usability burdens, uneven engagement, and variable acceptability across diverse youth populations [16]. Accordingly, feasibility studies in novel, youth-centered digital environments are a critical step toward identifying formats that young people can realistically and sustainably use.

One emerging ICT modality is the metaverse, broadly defined as a persistent, shared virtual space in which users interact through avatars within immersive (eg, virtual reality [VR]/mixed reality) or nonimmersive environments [17,18]. Unlike stand-alone VR or augmented reality applications that provide isolated, session-based experiences, metaverse environments are typically continuous, socially interactive spaces in which users can maintain identity, customize self-representation, and engage in repeated or ongoing interactions over time. These environments may include synchronous communication, spatialized presence, and customizable virtual settings, allowing users to experience a sense of copresence and embodiment beyond conventional video-based teleconferencing [19].

In youth mental health contexts, these features may be particularly relevant. Recent reviews of immersive and metaverse-based digital therapies suggest potential experiential advantages over conventional telepsychiatry; however, robust empirical evidence remains limited and heterogeneous [18]. Avatar-mediated interaction and the option to engage from a private physical location may lower the psychological threshold for help-seeking and support continued participation, as suggested by early metaverse-based or VR-enabled support initiatives [17,18,20]. In Japan, for example, emerging initiatives in some cities use VR-based support services for individuals with social withdrawal (Hikikomori) [20], exploring approaches to reduce stigma and facilitate access to support. The metaverse could extend these efforts internationally by offering an alternative environment in which young people can seek help discreetly and interactively while maintaining a sense of autonomy.

However, empirical evidence on the feasibility, acceptability, and ethical implementation of metaverse-based MHSs, such as psychiatric consultations for young people, remains limited, particularly in real-world clinical contexts. This study, therefore, aims to assess the feasibility of metaverse-based psychiatric consultations tailored for young people with mental health conditions, with a focus on user experience, usability, and implementation considerations. The findings will guide the development and implementation of future youth MHS, addressing key barriers to access and engagement.

## Methods

### Research Design Overview

This study employed a qualitative descriptive study to assess the feasibility of metaverse-based psychiatric consultations for youths with mental health conditions. Semistructured individual interviews were conducted following the consultations to explore participants’ experiences, perceived usability, and acceptability of the intervention.

### Study Participants or Data Sources

Participants were young people aged 16 to 25 years who self-identified as experiencing mental health conditions. The research team consisted of psychiatrists and mental health professionals with clinical experience in youth mental health care, as well as researchers with expertise in digital mental health and qualitative research. These backgrounds informed the study design, particularly the focus on feasibility, acceptability, and ethical considerations of metaverse-based psychiatric consultations.

Some participants were recruited from clinical settings where members of the research team were involved in their care. In such cases, the clinicians who had an existing therapeutic relationship with a participant did not conduct the postconsultation interviews and were not involved in the qualitative data analysis for that participant. This role separation was implemented to minimize potential power imbalances, social desirability bias, and undue influence on participants’ responses.

For participants recruited through educational institutions or social media, no prior relationship with the research team existed before study participation. All participants were informed that participation was voluntary, would not affect their clinical care or educational standing, and that they could withdraw at any time without consequences. These measures were taken to ensure ethical integrity and to support open and honest expression during the research process.

### Participant Recruitment

#### Recruitment Strategy

Participants were recruited between July and November 2023 through multiple channels, including flyer distribution at Yokohama University Hospital, nearby clinics, local universities, high schools, community support organizations, and announcements on social media platforms. Recruitment materials briefly described the study as an opportunity to experience a metaverse-based psychiatric consultation and to share feedback about the experience. Interested individuals contacted the research team directly and were screened for eligibility.

All participants received written and verbal explanations of the study procedures, potential risks, and the voluntary nature of participation. Written informed consent was obtained prior to study participation; for participants younger than 18 years, consent was also obtained from legal guardians in accordance with institutional review board requirements. Participants were compensated with a gift card valued at approximately US $35 (¥5000).

The target sample size was determined based on the exploratory aims of a qualitative descriptive feasibility design rather than statistical power considerations. A total of 33 individuals expressed interest in participation. Of these, 1 individual did not meet the inclusion criteria, 5 were lost to contact prior to providing informed consent, and 1 was unable to participate due to their condition on the scheduled day. Consequently, 26 participants completed the metaverse-based consultation and postconsultation interview.

Recruitment and data collection were concluded when thematic convergence was observed during qualitative analysis, and no substantially new themes emerged in later interviews, indicating that the sample size was sufficient for the aims of this feasibility study.

#### Inclusion and Exclusion Criteria

Eligible participants were individuals aged 16 to 25 years at the time of consent, who were aware of their mental health conditions and able to provide written informed consent in Japanese. Individuals currently receiving treatment at a psychiatric medical institution without permission from their primary physician were excluded from participation.

### Data Collection

#### Setting

The study was conducted in a meeting room on the Yokohama City University campus.

#### Baseline Assessment

As a baseline assessment, the participants completed a self-administered questionnaire consisting of 4 parts: (1) mental and physical conditions, (2) living conditions over the past 3 months, (3) internet usage, and (4) perceptions of mental health and psychiatry.

#### Metaverse Environment Setup and Technical Support

After the baseline assessment, the participants entered the metaverse environment under the guidance of technical staff, wearing VR goggles and using a controller. Since the participants were unfamiliar with metaverse operations, the technical staff provided detailed instructions for fitting the goggles, launching the application, and logging in.

For this study, experimental worlds and avatars were set up on commercially available metaverse applications, VRChat and Workrooms. VRChat is a social VR platform that allows users to create, share, and explore user-generated virtual worlds through customizable avatars, fostering social interaction and creative expression in immersive settings. In contrast, Workrooms is a virtual collaboration tool designed for professional and educational use, enabling users to hold virtual meetings in shared spaces with avatars and facilitating interactive discussions, presentations, and collaborative tasks. These applications provided structured and flexible virtual spaces suitable for the purposes of this study.

**Figure 1.**
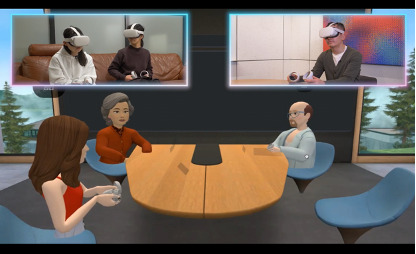
Overview of the metaverse-based psychiatric consultation process for youth with mental health conditions (N=26), conducted at Yokohama City University Hospital between July 2023 and November 2023. The image illustrates a participant and a clinician wearing head-mounted displays and engaging in interaction via avatars.

The virtual environments included 3 distinct spaces: an outer space environment, a hospital examination room, and a meeting room with selectable views of a beach or mountains. The avatars included both human and nonhuman fictional characters, providing participants with diverse ways of interacting and expressing themselves. Figure 1 illustrates one of the settings and avatars used in the study.

#### Metaverse Psychiatric Consultation

The metaverse consultations were conducted one-on-one in private virtual rooms with a psychiatrist, who also wore VR goggles and appeared as an avatar from a remote location. The psychiatrists included both male and female professionals with an average of 19 years of clinical experience.

Each consultation lasted 30 to 40 minutes and followed a semistructured framework resembling a standard initial psychiatric consultation. The consultation consisted of 5 phases. First, the psychiatrist introduced themselves via their avatar and explained the goals and structure of the session, allowing time to establish rapport and ensure that the participant felt comfortable in the virtual environment. Second, the psychiatrist reviewed the participant’s medical and life history, including prior diagnoses, treatments, daily routines, relationships, and environmental stressors. Third, the session focused on symptom observation and assessment, during which participants described their current mental health concerns while the psychiatrist observed verbal and nonverbal cues through avatar-mediated interaction. Fourth, tailored guidance and recommendations were provided, including coping strategies, lifestyle adjustments, and referrals to additional mental health resources when appropriate. Finally, the session concluded with a summary of the discussion and an opportunity for the participant to ask questions or provide feedback.

The consultations were designed to balance structure and flexibility, providing participants with a supportive environment to explore their mental health concerns in a novel virtual setting. During the session, the participants navigated the 3 distinct spaces within the metaverse environment alongside the psychiatrist and interacted via various types of avatars. Technical staff members were available to assist as needed, ensuring a seamless and accessible experience.

#### Postconsultation Interviews

After the metaverse consultation, participants were individually interviewed to explore their experiences and perceptions of the metaverse consultation. The semistructured interview guide used in this study was developed specifically for this project. An English version of the interview form is provided in Multimedia Appendix 1 for reference. The interviews explored several key areas, including the usability of the metaverse devices, any physical or psychological discomfort experienced, acceptance compared with face-to-face consultations, perceptions of individuals or situations in which metaverse consultations may be beneficial, and implementation-related requirements or concerns.

Following the participant interviews, the psychiatrists who conducted the consultations, along with members of the research team—including psychiatric social workers and a nurse—engaged in structured discussions regarding usability, expectations, and implementation challenges. These discussions addressed the functionality and user experience of the metaverse platform, technical considerations, perceived differences between metaverse and face-to-face consultations, compatibility with participant characteristics and symptom profiles, and additional observations noted during the study.

The reporting of this study conforms to the COREQ (Consolidated Criteria for Reporting Qualitative Research) guidelines (Checklist 1) [21].

### Data Analysis

#### Qualitative Analysis Strategy

The interview data were analyzed via thematic analysis following established qualitative research methodologies. First, all interviews were transcribed verbatim to ensure accuracy. The transcripts were then reviewed multiple times to facilitate familiarity with the data and identify potential patterns.

All interview transcripts were reviewed in full by the first author (MI), who performed line-by-line coding to identify meaningful units of text. Codes were generated inductively from the data and iteratively refined in consultation with 2 coauthors (JF and NT) to enhance credibility. Codes were clustered into categories, and higher-order themes were developed through repeated team discussions until consensus was reached.

Initial coding was conducted by systematically highlighting recurring phrases, concepts, and key statements. These codes were grouped into broader categories that represented emerging themes. To enhance rigor, the themes were independently reviewed and refined by multiple researchers to ensure consistency and reduce bias.

The analysis focused on 3 primary domains: the usability of the metaverse platform, the acceptance of metaverse consultations in comparison with face-to-face interactions, and the identification of participant characteristics or conditions for which metaverse consultations may offer particular advantages, such as social anxiety or sensory sensitivities.

#### Methodological Integrity

To ensure trustworthiness, we maintained an audit trail of coding decisions, documenting how codes were generated, refined, and grouped into themes. Triangulation was achieved through iterative discussions among the research team, allowing for verification and consensus in data interpretation. Representative quotations were included in the *Results* section and labeled with participant numbers to illustrate key themes and to provide transparency between the participants’ voices and the researchers’ interpretations.

Data collection was concluded when data saturation was reached, defined as the point at which no new themes or insights emerged from the interviews. No substantially new themes emerged in the latter part of the interviews. Thus, we consider the sample size sufficient for the exploratory aims of this qualitative feasibility study.

This study was conducted and reported in accordance with the APA Journal Article Reporting Standards for qualitative research [22].

### Ethical Considerations

This study was conducted in accordance with the Declaration of Helsinki and the Ethical Guidelines for Life Science and Medical Research Involving Human Subjects, established by the Ministry of Education, Culture, Sports, Science and Technology and the Ministry of Health, Labour and Welfare of Japan (partially revised in 2023). The study protocol was approved by the institutional review board of Yokohama City University, Japan (approval number F230400055). Written informed consent was obtained from all participants prior to their inclusion in the study; for participants younger than 18 years, additional consent was obtained from their legal guardians along with participant assent, where applicable. To ensure privacy and confidentiality, all study data were deidentified during handling and analysis, and interview transcripts were meticulously reviewed to remove any personal identifiers. Participants were compensated for their time with a gift card valued at approximately US $35 (¥5000). Regarding the visual materials, this manuscript includes images of staff members demonstrating the metaverse consultation environment. All identifiable individuals in these images provided explicit written informed consent for the publication of their likeness, and the relevant consent forms are available for verification upon request.

Since this study was conducted as an observational study, clinical trial registration was not applicable.

## Results

The results presented below were derived through thematic analysis of postconsultation interviews, identifying common patterns across participants’ responses.

### Baseline Characteristics

A total of 26 young people were assigned from a pool of 33 volunteers. One individual was excluded for not meeting the inclusion criteria, 5 lost contacts before providing informed consent, and 1 could not participate because of his condition on the day. Table 1 summarizes the baseline characteristics, while Table 2 presents the results of the baseline assessments of the participants. The mental and physical conditions of the participants varied, with many expressing difficulties in social interactions, feelings of isolation, and discomfort in seeking support from others. Most participants reported frequent internet use, although their levels of social engagement and self-care practices differed. All participants owned internet-enabled devices, and most used the internet daily. However, some participants acknowledged neglecting essential tasks due to internet use, whereas others reported difficulties in limiting their internet usage. The participants’ initial impressions of the metaverse also varied. While only a minority had extensive knowledge or prior experience with the metaverse, many expressed curiosity. Their views on associating with individuals who had a history of psychiatric treatment ranged widely, with some expressing comfort and acceptance, whereas others showed apprehension or stigma toward mental health issues.

**Table 1.** Baseline demographic and clinical characteristics of youth participants with mental health conditions (N=26) in an observational feasibility study of metaverse-based psychiatric consultation conducted at Yokohama City University between July 2023 and November 2023.

Characteristic	Description
Age (y), mean (range)	19.9 (16-25)
Gender	
Male	15
Female	7
Nonbinary	3
No response	1
Recruitment sources	Yokohama City University Health Management Center, medical students and junior residents, outpatient of University Hospital and neighborhood clinics, participants from other survey of our research team, neighborhood private high school, Facebook
History of psychiatric treatment	
Yes	22
No	4

**Table 2.** Summary of baseline clinical assessments, including psychological symptoms and functional status, of youth participants (N=26) prior to the metaverse-based psychiatric consultation.

Aspect of assessment	Summary of findings
1. Physical and psychological condition	
Perceived health issues	Most participants (21/26, 80%) indicated concerns about their physical or mental health, with common issues including insomnia, social anxiety, general anxiety, depression, learning disabilities, difficulty speaking with others, and lack of motivation.
Comfort being in social situations	Nearly two-thirds (18/26, 68%) reported discomfort being in front of others, and 64% (17/26) felt uncomfortable talking with strangers, indicating significant social anxiety.
Help-seeking behavior	Approximately 65% (17/26) reported that they seek advice when facing problems, primarily consulting family, friends, teachers, or medical staff.
Sense of social connection	Mixed responses: 50% (13/26) reported sometimes feeling a lack of social connections, while others felt relatively neutral or disagreed with feelings of isolation.
Feelings of exclusion and isolation	Most participants (20/26, 77%) felt neither excluded nor isolated, suggesting a generally stable sense of belonging, despite some individual cases of feeling isolated or left out.
2. Living condition of the past 3 months	
Frequency of going out	Most participants went out frequently, with 73% (19/26) going out at least once a week, while 27% (7/26) went out less frequently.
Conversations with family members	The majority (23/26, 88%) reported daily conversations with family, indicating strong familial interaction.
Conversations with nonfamily members	Over half (20/26, 77%) had conversations with nonfamily members at least weekly, suggesting moderate social interaction outside the family.
Ability to maintain a regular routine	Only 27% (7/26) of participants maintained a regular routine daily or several times per week, with the remainder struggling to do so consistently.
Sufficient sleep	Approximately half (13/26, 50%) reported receiving sufficient sleep almost daily or several times per week, while others reported inconsistent sleep.
Frequency of bathing or showering	Nearly all participants (24/26, 92%) bathed or showered daily, indicating consistent personal hygiene practices.
Concentration on work or studies	Only 27% (7/26) could concentrate on work or studies consistently (daily or multiple times per week), with others facing challenges in concentration.
Engagement in nonwork/study activities	Most participants (16/26, 61%) engaged in hobbies, sports, or social activities at least once a week, while others were less active.
3. Internet usage	
Access to internet-enabled devices	All participants (26/26, 100%) owned internet-enabled devices, such as computers, smartphones, or gaming consoles.
Frequency of internet use	Nearly all participants reported daily internet use, indicating high internet engagement.
Neglect of responsibilities due to internet use	A majority (19/26, 73%) sometimes or always neglected responsibilities due to internet use, with only one participant reporting no impact.
Making new connections online	Around half (12/26, 46%) reported occasionally or frequently making new connections online, while 27% (7/26) never made new connections through the internet.
Difficulty reducing internet use	Nearly half (12/26, 46%) indicated difficulty reducing internet use, with the remainder having little to no issues in managing their usage.
Familiarity with the metaverse	Knowledge about the metaverse varied, with 46% (12/26) reporting some familiarity, while the rest indicated limited or no knowledge.
Experience with the metaverse	Only a small portion (6/26, 23%) had direct experience with the metaverse, indicating it was relatively new to most participants.
4. Attitudes toward mental health and psychiatry	
Friendship with individuals who have seen a psychiatrist	Most participants (23/26, 88%) expressed openness, agreeing that they could become close friends with someone who has sought psychiatric care.
Trust in individuals with a history of psychiatric hospitalization	A majority (19/26, 73%) believed that individuals with past psychiatric hospitalization are just as trustworthy as others.
Perception of psychiatric treatment as a personal failing	Most participants (22/26, 85%) disagreed with the notion that seeking psychiatric help is a sign of personal failure.
Reluctance to hire individuals with psychiatric history	Over half (17/26, 65%) felt that many people would be reluctant to hire someone with a history of psychiatric treatment, reflecting a perceived societal stigma.
Willingness to date someone with a history of psychiatric treatment	Around half (14/26, 54%) were neutral or positive about dating someone with a psychiatric history, while 27% (7/26) expressed reluctance.

### Interviews

**Figure 2.**
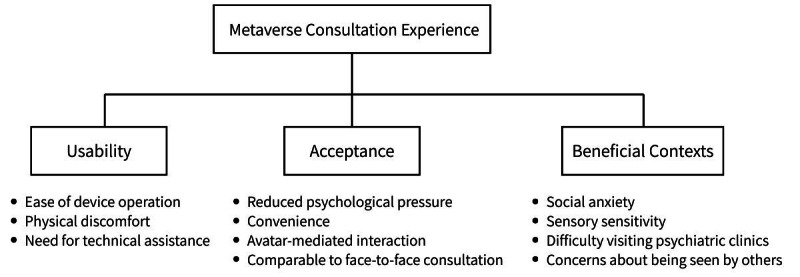
Thematic map of participants’ experiences with metaverse-based psychiatric consultations (N=26) conducted at Yokohama City University Hospital between July 2023 and November 2023. The map illustrates the relationships among the key themes identified through thematic analysis of postconsultation interviews: usability of the metaverse device, acceptance of metaverse consultations, and contexts in which metaverse consultations may be particularly beneficial for young people with mental health conditions.

Thematic analysis was used to identify patterns and themes from participants’ postconsultation interviews. A thematic map summarizing the relationships among the identified themes is presented in Figure 2.

#### Usability of the Metaverse Device

Participants expressed a range of views regarding usability. Approximately 46% reported positive experiences, describing the device as intuitive or comparable to gaming interfaces. About 23% expressed neutral views, noting that while manageable for short sessions, prolonged use might be physically demanding. In contrast, 31% reported negative experiences, primarily related to physical discomfort, device fit, or technical complexity.

Participants who reported positive experiences noted that they “quickly got used to the controls” (Participant 10, 19, 20), and found the device “easy to use due to regular use of gaming devices” (Participant 9, 13, 16, 22). Optimism for future accessibility was evident in comments such as “I would like to use it when it becomes easier” (Participant 4) and “more affordable” (Participant 23, 24).

Participants with neutral views indicated that although they adapted to the operations, more complex tasks might require assistance. One participant commented,

I feel comfortable for short sessions, but longer sessions might be tough with these devices.

[Participant 4]

Those expressing negative views raised concerns about the device’s physical fit and comfort. Nine participants experienced issues such as the goggles feeling “too tight” (Participant 18), “too loose and slipping” (Participant 13, 14)*,* or challenging to fit over glasses. Five participants reported mild physical discomfort, including VR-related discomfort or head heaviness, although no psychological discomfort was noted. A participant remarked, “It might be difficult to connect and operate remotely without staff assistance” (Participant 18), highlighting barriers to independent use.

#### Acceptance of the Metaverse Consultation

Responses regarding the acceptance of metaverse consultations revealed a range of perspectives.

Thirty-eight percent of participants expressed a preference for metaverse consultations over face-to-face sessions, citing benefits such as reduced psychological pressure and convenience. The comments included, “It’s a big help not having to leave home when I’m feeling too unwell to go out” (Participant 12) and “Meeting a new doctor is less nerve-wracking through an avatar” (Participant 3). Another participant noted,

I don’t have to endure the intimidating atmosphere of psychiatric institutions or the doctor’s imposing presence.

[Participant 19]

Another 38% of participants expressed neutral responses, describing the content and quality of metaverse consultations as comparable to face-to-face consultations. Many noted that their preference would depend on their condition at the time. For example, one participant remarked,

I prefer face-to-face if I can go out, but there are times I’d rather use the metaverse.

[Participant 18]

Others suggested that

The metaverse would be preferable if issues such as cost, ease of access, and privacy were addressed.

[Participant 24]

The remaining 24% preferred face-to-face consultations, emphasizing the reassurance of observing facial expressions and body language. One participant shared,

If I can’t see their face, I worry whether they’re truly understanding or accurately interpreting what I want to convey.

[Participant 4]

Another added,

For personal and serious discussions, I want to see the person face-to-face.

[Participant 8]

Participants also shared insights into specific scenarios where metaverse consultations might be beneficial. One participant stated,

It’s ideal for people like me who are self-conscious about being seen. I can focus more on the consultation and express what I truly want to say.

[Participant 9]

Another remarked,

It’s helpful for those who find it challenging to visit psychiatric clinics.

[Participant 1]

#### Keywords and Concerns for Metaverse Consultations

Participants highlighted several key requirements and concerns regarding implementation. Security and privacy were frequently emphasized, particularly the need for a secure and private consultation environment. The participants requested assurances that psychiatrists understood them accurately. One participant commented,

I don’t want to show my face, but if the avatar’s facial expressions and body movements are synchronized with reality, it feels more reassuring than a phone call.

[Participant 1]

Concerns about information security for private consultations were also raised.

Customizability of avatars and virtual spaces was another recurring theme. Participants frequently emphasized the importance of customizable settings, using terms such as “relaxing,” “free from distractions,” and “self-selectable.” Twelve participants expressed a preference for avatars that felt authentic to them, with one stating,

choosing my avatar is a way of expressing myself, and it makes me feel more comfortable in the consultation.

[Participant 10]

Preferences for human avatars (6 participants) and nonhuman characters (6 participants) were evenly split.

#### Psychiatric Team’s Perspective

The psychiatric team provided valuable insights into the potential benefits and challenges of metaverse consultations.

Team members noted that individuals with sensory hypersensitivity, such as those with traits associated with autism spectrum disorder, appeared to focus more effectively in the immersive VR environment. They also reported that individuals with significant social anxiety or tension seemed to relax more quickly, enabling broader and deeper conversations.

At the same time, concerns were raised regarding the suitability of metaverse consultations for participants with pronounced symptoms, as certain clinical cues may be less observable in virtual settings. Suggestions included simplifying metaverse operations for easier access and expanding the range of avatars and room settings to allow for more personalized consultations. They also recommended developing diagnostic support tools to enhance the consultation experience.

## Discussion

### Principal Findings

This study evaluated the feasibility and user experience of metaverse-based psychiatric consultations for young people with mental health conditions. The findings demonstrated that, in this study, such consultations are technically feasible and safe, with no major adverse events. Participants reported high levels of engagement, and their postexperience feedback highlighted the unique advantages of avatar-mediated interaction. While some physical discomfort was noted, the overall acceptance of metaverse consultations suggests their potential as a complementary modality in youth mental health care.

### Interpretations of Findings

Our results identified specific groups of young people who showed a strong affinity for metaverse consultations, including those with social anxiety, sensory sensitivities (eg, autistic traits), difficulty leaving home due to mental illness, concerns about stigma, and discomfort with their physical appearance. For these individuals, avatar-based interaction lowered psychological barriers and provided a sense of safety and autonomy. These findings are consistent with existing literature showing that digital interventions can enhance help-seeking among youth when designed to offer privacy, flexibility, and user control [9,15,20].

The immersive VR environment was particularly appreciated by participants with sensory sensitivities, as one participant remarked,

After experiencing the metaverse consultation, I felt I truly understood what a consultation was meant to be for the first time.

[Participant 2]

This finding may be interpreted through the lens of presence theory, which suggests that immersive environments can enhance users’ sense of “being there,” potentially facilitating emotional engagement and attentional focus [23].

The ability to interact through avatars in a spatialized environment may also support a form of digital embodiment, enabling participants to regulate interpersonal distance and self-presentation more flexibly than in face-to-face or video-based consultations. This capacity to modulate proximity and representation aligns with findings from embodiment research, which suggest that virtual body ownership and perspective-taking can influence affective and social responses [24]. Such mechanisms may partially explain why some participants reported feeling more relaxed and able to engage in deeper conversations within the metaverse setting.

On the other hand, previous studies caution that VR may also induce discomfort in some individuals with autism spectrum disorder [24]. This duality highlights the need for individualized approaches and flexible options.

In terms of accessibility, our findings revealed operational challenges such as the need for technical support, device heaviness, and VR-related discomfort. These barriers align with broader concerns regarding the digital divide and unequal access to advanced immersive technologies, particularly among young people with mental health conditions. Previous research has demonstrated that individuals with mental ill health, including socioeconomically and digitally marginalized youth, may be limited or nonusers of digital interventions despite device access, suggesting that skills, motivation, and confidence can constitute significant barriers to digital engagement [25,26]. Access to high-end VR hardware may require financial, technological, and spatial resources that are not evenly distributed, potentially exacerbating existing disparities in MHS utilization.

From a technology acceptance perspective, perceived ease of use and perceived usefulness are central determinants of user adoption in health technologies [26]. When devices are physically uncomfortable or operationally complex, perceived ease of use may decline, thereby reducing users’ willingness to engage with the platform. Therefore, simplifying hardware requirements and offering lighter, cross-platform alternatives may be essential to ensure equitable and scalable implementation of metaverse-based MHSs. This underscores the importance of exploring less resource-intensive alternatives, such as “light metaverse” platforms that are accessible via widely available devices, including smartphones or personal computers. Expanding metaverse options beyond VR goggles could help reach a wider population and improve inclusivity [18].

In interpreting these findings, it is also important to situate metaverse-based consultations in relation to existing digital mental health modalities, particularly video-based teleconferencing, which is currently the most widely used form of telepsychiatry. Teleconferencing has demonstrated effectiveness in increasing access to care and reducing geographical barriers [27,28]. However, because it typically relies on real-time videoconferencing, it retains key interpersonal elements of face-to-face encounters (eg, visual and vocal cues, facial interaction, and eye contact), while also introducing heightened self-presentation and appearance-related attentional demands [29]. In contrast, metaverse-based consultations have been proposed to offer distinct experiential affordances through avatar-mediated interaction and immersive environments [18]. For some participants, these features appeared to reduce psychological barriers associated with being seen, judged, or scrutinized, thereby facilitating disclosure and engagement. At the same time, the use of VR technology introduced additional usability burdens, including physical discomfort, device complexity, and the need for technical support, which are less prominent in conventional teleconferencing. These findings suggest that metaverse-based consultations should not be viewed as a replacement for teleconferencing, but rather as a complementary modality that may be particularly valuable for specific subgroups of young people for whom video-based interaction remains challenging. Future research directly comparing immersive metaverse approaches with standard teleconferencing is needed to clarify relative advantages, limitations, and appropriate indications within stepped or hybrid models of youth mental health care.

Participants also emphasized the value of choice in how they receive mental health support. The concept of choice emerged consistently in participants’ responses, highlighting their desire for flexibility and personalization. The ability to choose avatars and participate in consultations from private spaces fostered a greater sense of agency. This aligns with prior research emphasizing the importance of autonomy and co-designed services in engaging youth with mental health needs [6,17,30,31].

Ethical challenges remain. While our study obtained consent in person, widespread adoption of metaverse consultations will require mechanisms to ensure informed consent, protect privacy, and uphold safety in fully virtual settings [32]. Addressing these ethical and operational hurdles is essential for translating the potential of virtual environments into sustainable clinical practice.

### Strengths and Limitations

#### Strengths

This is the first observational trial to explore the feasibility of psychiatric consultations in a metaverse setting, offering novel insights into how digital environments can be leveraged to support youth mental health. The study benefited from a youth-centered design, the involvement of experienced psychiatrists, and the incorporation of diverse participant feedback. This design enabled a nuanced understanding of how metaverse-based consultations may meet the psychological and logistical needs of underserved youth populations, including those with interpersonal anxiety, sensory sensitivities, or barriers to in-person care.

#### Limitations

Several limitations should be noted. First, the sample size was modest, and participants were primarily recruited from urban or institutional settings, which may limit the generalizability of the findings to broader or more diverse populations. Second, the study focused solely on avatar-based communication within VR platforms, without evaluating broader features of the metaverse, such as social engagement spaces, gamified environments, or decentralized user governance structures such as DAOs. Third, populations such as nonnative speakers or individuals with speech impairments were not included, which may limit the inclusivity of the findings. Future studies should explore comparative modalities (eg, light vs heavy metaverse), include more diverse participants, and assess additional functional elements of digital environments to further understand the potential of metaverse-based mental health support.

### Implications and Conclusions

This study provides early evidence that metaverse-based psychiatric consultations are a feasible and acceptable approach for supporting young people with mental health conditions. The innovation of this study lies in its focus on avatar-mediated psychiatric consultation within a metaverse environment, moving beyond conventional telepsychiatry models by examining how immersive, customizable virtual spaces may influence user experience and engagement. Unlike prior digital mental health studies that primarily evaluate video- or text-based teleconferencing, this study highlights the distinct role of immersive and self-representational features—such as avatars and virtual environments—in potentially reducing psychological barriers, enhancing autonomy, and supporting help-seeking among youth who experience anxiety, stigma, or discomfort with face-to-face care. By identifying specific subgroups of youth for whom metaverse-based consultations may be particularly beneficial, this study contributes to the emerging field of youth digital mental health by offering practical insights into when and for whom immersive technologies may add value beyond existing modalities.

From a real-world perspective, metaverse-based psychiatric consultations may serve as a complementary access point within stepped or hybrid care models, particularly for young people who face psychological or logistical barriers to in-person or video-based services. However, careful consideration of usability, digital equity, and ethical safeguards will be essential to ensure responsible and scalable implementation. Future research should compare immersive metaverse approaches with conventional teleconferencing, examine long-term clinical outcomes, and explore scalable implementation strategies to support equitable and youth-centered mental health care across diverse settings.

## Supplementary material

10.2196/83688 Multimedia Appendix 1 Interview guide that is used for semistructured interview for participants.

10.2196/83688 Checklist 1 COREQ checklist.

## Abbreviations

COREQ: Consolidated Criteria for Reporting Qualitative Research
ICT: information and communication technology
MHS: mental health service
VR: virtual reality

## References

[1] Lu W (2019). Adolescent depression: national trends, risk factors, and healthcare disparities. Am J Health Behav.

[2] Kowalchuk A, Gonzalez SJ, Zoorob RJ (2022). Anxiety disorders in children and adolescents. Am Fam Physician.

[3] Racine N, McArthur BA, Cooke JE, Eirich R, Zhu J, Madigan S (2021). Global prevalence of depressive and anxiety symptoms in children and adolescents during COVID-19: a meta-analysis. JAMA Pediatr.

[4] Kessler RC, Angermeyer M, Anthony JC (2007). Lifetime prevalence and age-of-onset distributions of mental disorders in the World Health Organization’s World Mental Health Survey Initiative. World Psychiatry.

[5] Mental health of adolescents. World Health Organization.

[6] Aguirre Velasco A, Cruz ISS, Billings J, Jimenez M, Rowe S (2020). What are the barriers, facilitators and interventions targeting help-seeking behaviours for common mental health problems in adolescents? A systematic review. BMC Psychiatry.

[7] Radez J, Reardon T, Creswell C, Lawrence PJ, Evdoka-Burton G, Waite P (2021). Why do children and adolescents (not) seek and access professional help for their mental health problems? A systematic review of quantitative and qualitative studies. Eur Child Adolesc Psychiatry.

[8] Bui AL, Ball AM, Ko LK, Ng M, Rivara FP, Coker TR (2025). Foregone preventive care and unmet mental healthcare needs among U.S. youth. Am J Prev Med.

[9] McGorry PD, Mei C, Chanen A, Hodges C, Alvarez-Jimenez M, Killackey E (2022). Designing and scaling up integrated youth mental health care. World Psychiatry.

[10] Roulston CA, Ahuvia I, Chen S, Fassler J, Fox K, Schleider JL (2025). “My family won’t let me.” Adolescent-reported barriers to accessing mental health care. J Res Adolesc.

[11] Wahdi AE, James C, Nadhira DA, Fine SL (2025). Barriers and facilitators of seeking help for mental health challenges among adolescents across 13 countries: a qualitative investigation. J Adolesc Health.

[12] Ma KKY, Anderson JK, Burn AM (2023). Review: school-based interventions to improve mental health literacy and reduce mental health stigma - a systematic review. Child Adolesc Ment Health.

[13] Seedaket S, Turnbull N, Phajan T, Wanchai A (2020). Improving mental health literacy in adolescents: systematic review of supporting intervention studies. Trop Med Int Health.

[14] McGorry P, Gunasiri H, Mei C, Rice S, Gao CX (2024). The youth mental health crisis: analysis and solutions. Front Psychiatry.

[15] Hollis C (2022). Youth mental health: risks and opportunities in the digital world. World Psychiatry.

[16] Opie JE, Vuong A, Welsh ET (2024). Outcomes of best-practice guided digital mental health interventions for youth and young adults with emerging symptoms: part I. A systematic review of socioemotional outcomes and recommendations. Clin Child Fam Psychol Rev.

[17] Navas-Medrano S, Soler-Dominguez JL, Pons P (2023). Mixed reality for a collective and adaptive mental health metaverse. Front Psychiatry.

[18] Buragohain D, Khichar S, Deng C, Meng Y, Chaudhary S (2025). Analyzing metaverse-based digital therapies, their effectiveness, and potential risks in mental healthcare. Sci Rep.

[19] Oh HJ, Kim J, Chang JJC, Park N, Lee S (2023). Social benefits of living in the metaverse: the relationships among social presence, supportive interaction, social self-efficacy, and feelings of loneliness. Comput Human Behav.

[20] The world’s first hikikomori research lab @ Kyushu University. Kyushu University.

[21] Tong A, Sainsbury P, Craig J (2007). Consolidated criteria for reporting qualitative research (COREQ): a 32-item checklist for interviews and focus groups. Int J Qual Health Care.

[22] Levitt HM, Bamberg M, Creswell JW, Frost DM, Josselson R, Suárez-Orozco C (2018). Journal article reporting standards for qualitative primary, qualitative meta-analytic, and mixed methods research in psychology: the APA Publications and Communications Board task force report. Am Psychol.

[23] Slater M, Sanchez-Vives MV (2016). Enhancing our lives with immersive virtual reality. Front Robot AI.

[24] Satu P, Minna L, Satu S (2025). Immersive VR assessment and intervention research of individuals with neurodevelopmental disorders is dominated by ASD and ADHD: a scoping review. Rev J Autism Dev Disord.

[25] Piers R, Williams JM, Sharpe H (2023). Review: can digital mental health interventions bridge the “digital divide” for socioeconomically and digitally marginalised youth? A systematic review. Child Adolesc Ment Health.

[26] Holden RJ, Karsh BT (2010). The technology acceptance model: its past and its future in health care. J Biomed Inform.

[27] Mucic D, Shore J, Hilty DM (2023). Overview of the World Psychiatric Association telepsychiatry global guidelines. J technol behav sci.

[28] Duffy CMC, Benotsch EG (2025). Nonverbal behavior in telehealth visits: a narrative review. Patient Educ Couns.

[29] Gibson K (2021). What Young People Want from Mental Health Services: A Youth Informed Approach for the Digital Age.

[30] Hetrick SE, Bailey AP, Smith KE (2017). Integrated (one-stop shop) youth health care: best available evidence and future directions. Med J Aust.

[31] Raja US, Al-Baghli R (2025). Ethical concerns in contemporary virtual reality and frameworks for pursuing responsible use. Front Virtual Real.

[32] Bouchard S (2017). Virtual reality exposure therapy for social anxiety disorder: a randomized controlled trial. J Consult Clin Psychol.

