# Supplementary material for: Metaverse-Based Psychiatric Consultation for Youths With Mental Health Conditions: Qualitative Descriptive Feasibility Study
Source: JMIR XR Spat Comput. 2026 May 5;3:e83688. doi: 10.2196/83688 (PMC13202498; doi:10.2196/83688)
Supplement: Multimedia Appendix 1 [file xr-v3-e83688-s001.pdf]

Supplementary File 2. Semi-Structured Interview Guide

This semi-structured interview was developed specifically for this study. It was conducted after the Metaverse consultation sessions to explore participants' experiences, acceptance, and perceptions of challenges related to the use of Metaverse-based mental health consultations. The interviews lasted approximately 20 minutes.

Semi-Structured Interview Form

Following the content implementation, a semi-structured interview is conducted based on the following questions. The interview focuses on topics related to general impressions of the Metaverse and digital content as well as participants' experiences with metaverse psychiatric consultations. Over approximately 20 minutes of discussion, the interview aims to explore participants' acceptance, usability, and future challenges related to the simulated metaverse consultation process.

1. Feasibility of Metaverse-Based Mental Health Services (Usability, willingness for future use, etc.)
2. Did you find the system easy to use?
3. Did you experience any physical discomfort, such as VR sickness?
4. Would you like to use this service of metaverse psychiatric consultations in the future?
5. Would you recommend this service to others?
6. Compatibility with Metaverse-Based Mental Health Services (Interpersonal anxiety, preferences for digital tools, etc.)
7. Do you think using PCs, smartphones, or gaming devices poses any harm?
8. Do you find communication features, such as chat functions on digital platforms, convenient?
9. Do you feel any anxiety about consulting in the Metaverse?
10. Which do you prefer—face-to-face consultations or Metaverse-based consultations?
11. Additional Questions
12. Did you understand the content provided in the Metaverse?
13. Do you have any suggestions or preferences regarding the platform?
14. Do you have any suggestions or preferences regarding the avatars?
15. Are there any other questions or topics about the Metaverse that you would like to ask or learn more about?
16. Please share any additional comments, opinions, or feedback.
