# Supplementary material for: Metaverse-Based Psychiatric Consultation for Youths With Mental Health Conditions: Qualitative Descriptive Feasibility Study
Source: JMIR XR Spat Comput. 2026 May 5;3:e83688. doi: 10.2196/83688 (PMC13202498; doi:10.2196/83688)
Supplement: Checklist 1 [file xr-v3-e83688-s002.pdf]

COREQ Checklist (Consolidated Criteria for Reporting Qualitative Research)

This checklist was completed for the study 'Feasibility of Psychiatric Consultation by Metaverse for Youths with Mental Health Conditions'. It is based on the 32-item COREQ (Tong et al., 2007) framework.

Domain 1: Research team and reflexivity

1. Interviewer/facilitator: Which author(s) conducted the interviews or focus groups? *Mi and JF conducted the interviews.*
2. Credentials: What were the researcher's credentials (eg, PhD, MD)? *Among the research team, six held MDs, five of whom also had PhDs. The remaining two authors held university degrees and were licensed professionals: one nurse and one psychiatric social worker.*
3. Occupation: What was their occupation at the time of the study? *At the time of the study, one researcher was a psychiatric social worker, one was a nurse, and the others were practicing medical doctors.*
4. Gender: Was the researcher male or female? *The research team consisted of five females and three males.*
5. Experience and training: What experience or training did the researcher have? *Experience and training: What experience or training did the researcher have? All researchers had completed national clinical training in their respective professions. Those holding PhDs also had prior experience conducting clinical research in psychiatry and other medical fields.*
6. Relationship established: Was a relationship established prior to study commencement? *Yes. A relationship was established prior to study commencement through initial recruitment explanations and informed consent discussions.*
7. Participant knowledge of the interviewer What did the participants know about the researcher? *A relationship was established prior to study commencement through initial recruitment explanations and informed consent discussions.*
8. Interviewer characteristics: What characteristics were reported about the interviewer/facilitator? *The interviewers were psychiatrists with an average of more than 12 years of clinical experience.*

Domain 2: Study design

9. Methodological orientation and theory: What methodological orientation was stated to underpin the study? *Thematic analysis was used to analyze the interview data. This approach was chosen for its flexibility in identifying patterns across participant experiences.*
10. Sampling: How were participants selected (eg, purposive, convenience, snowball)? *Participants were recruited purposively from clinics, universities, high schools, support*

groups, and social media to capture a diverse group of young people experiencing mental health challenges.

11. Method of approach: How were participants approached (eg, face-to-face, telephone, mail, email)? *Potential participants were approached in person through collaborating institutions (e.g., Yokohama University Hospital and affiliated clinics), and via online recruitment through social media.*

12. Sample size: How many participants were in the study? *A total of 26 participants were included in the study.*

13. Non-participation: How many people refused to participate or dropped out? Reasons? *Of 33 initial volunteers, one was excluded for not meeting inclusion criteria, five lost contact before informed consent, and one withdrew due to health issues on the study day.*

14. Setting of data collection: Where was the data collected? *Data were collected in a meeting room at Yokohama University campus, where participants experienced the Metaverse consultation and then completed the interviews.*

15. Presence of non-participants: Was anyone else present besides the participants and researchers? *Only participants and interviewers were present during the interviews. Technical staff were available during the Metaverse consultation but did not join the interview sessions.*

16. Description of sample: What are the important characteristics of the sample (eg, demographic data, date)? *Participants were aged 16–25 years, included both male and female participants, and represented individuals with varying levels of prior psychiatric service use. Baseline characteristics are presented in Table 1 (see Supplementary File 1).*

17. Interview guide: Were questions, prompts, guides provided by the authors? Was it pilot tested? *A semi-structured interview guide was developed specifically for this study. The English version is provided as Supplementary File 1.*

18. Repeat interviews: Were repeat interviews carried out? If yes, how many? *No repeat interviews were conducted.*

19. Audio/visual recording: Did the research use audio or visual recording to collect the data? *No audio or video recordings were made. Instead, detailed field notes were taken during and immediately after the interviews to capture participant responses as accurately as possible.*

20. Field notes: Were field notes made during and/or after the interview or focus group? *Field notes were taken by interviewers during and after the interviews to capture contextual impressions.*

21. Duration: What was the duration of the interviews or focus group? *Each interview lasted approximately 20 minutes.*

22. Data saturation: Was data saturation discussed? *Data collection continued until all recruited participants completed interviews; saturation was considered when recurring themes emerged across responses.*

23. Transcripts returned: Were transcripts returned to participants for comment and/or correction? *Transcripts were not returned to participants for comment or correction.*

Domain 3: Analysis and findings

24. Number of data coders: How many data coders coded the data? *All transcripts were coded by a single researcher (MI). To enhance credibility, the coding framework and emerging themes were regularly discussed with other team members (JF, NT), who provided feedback and confirmed the interpretations.*

25. Description of the coding tree: Did authors provide a description of the coding tree? *A coding framework was developed inductively from the data. Codes were grouped into categories reflecting emerging themes, which were then organized into three overarching domains: (1) usability of the Metaverse platform, (2) acceptance of Metaverse consultations, and (3) participant characteristics associated with high affinity for Metaverse consultations.*

26. Derivation of themes: Were themes identified in advance or derived from the data? *Themes were derived inductively from the data through iterative coding and team discussions.*

27. Software: What software, if applicable, was used to manage the data? *Data were analyzed manually without the use of qualitative data analysis software.*

28. Participant checking: Did participants provide feedback on the findings? *Participants did not provide feedback on the findings.*

29. Quotations presented: Were participant quotations presented to illustrate the themes/findings? Was each quotation identified? *Representative quotations were provided in the Results section to illustrate themes, but they were not labeled with participant numbers; instead, anonymized direct quotations were presented without identifiers.*

30. Data and findings consistent: Was there consistency between the data presented and the findings? *There was a clear alignment between the presented data (quotations) and the findings described.*

31. Clarity of major themes: Were major themes clearly presented in the findings? *Major themes are clearly presented in the Results, including usability, acceptance, and key requirements for Metaverse consultations.*

32. Clarity of minor themes: Is there a description of diverse cases or discussion of minor themes? *Minor themes, such as concerns about privacy, avatar preferences, and physical discomfort, are also reported to capture the breadth of participant perspectives.*
